# Employee–AI collaboration empowers mentor networks to enhance employee creativity: a knowledge-management perspective

**DOI:** 10.3389/fpsyg.2026.1750869

**Published:** 2026-02-24

**Authors:** Miaomiao Li, Yang Yu, Jielin Yin

**Affiliations:** Business School, Beijing Information Science and Technology University, Beijing, China

**Keywords:** employee–AI collaboration, employee creativity, knowledge management, mentor network, tacit knowledge acquisition

## Abstract

This study investigates the impact of mentor network strength on employee creativity, as well as the mediating role of tacit knowledge acquisition and the moderating role of employee-AI collaboration. A three-wave survey was conducted among 523 employees from Chinese enterprises, and the proposed hypotheses were tested using confirmatory factor analysis, hierarchical regression, structural equation modeling, and bootstrapping. The results reveal the following: mentor network strength can promote employee creativity by enhancing cognitive tacit knowledge acquisition and skill-based tacit knowledge acquisition. Specifically, the mediating effect of skill-based tacit knowledge acquisition is more significant than that of cognitive tacit knowledge acquisition. Moreover, employee-AI collaboration positively moderates the relationship between mentor network strength and cognitive and skill-based tacit knowledge acquisition. The positive effect of mentor network strength on employee creativity via cognitive tacit knowledge acquisition and skill-based tacit knowledge acquisition is more significant when there is a higher degree of employee-AI collaboration. From a knowledge management perspective, this study fills a gap by exploring the mechanism by which mentor network strength affects employee creativity and offers a new perspective for interpreting the factors influencing employee creativity. The findings facilitate a deeper understanding of the synergistic effects of human-AI collaboration and interpersonal relationships on employee creativity, providing guidance to help organizations promote intelligent transformation.

## 1 Introduction

With rapid advancements in new technologies and increasingly fierce market competition, innovation emerges as a primary driver of development. Employees, as key contributors to organizational innovation (Song et al., 2019), play a central role in driving enterprise innovation and creativity. The knowledge, resources, and capabilities embedded in employees are a vital source of sustainable competitive advantage for enterprises (Wang and Chang, 2017). To some extent, the innovation ability of employees represents the innovation level of enterprises (Rujie et al., 2019). Employee creativity is defined as the generation of novel, useful ideas about products, services, and processes at work, which is essential for an organization’s survival and effectiveness (Yongyue et al., 2020). Employee creativity is an important driver of innovation, growth, and social development and a key factor helping organizations to adapt and develop in dynamic environments (Perry-Smith, 2006). Thus, how to stimulate employee creativity within organizations is a common concern among both researchers and managers (Bos-Nehles et al., 2017).

Effective interpersonal interactions are conducive to stimulating and sustaining employee innovation (Wasserman and Faust, 1994). Mentorship is considered an effective solution (Bang and Reio Jr, 2017), and it has been used by an increasing number of firms for new employee development, executive pipeline building, knowledge transfer, and human capital enhancement (Chen and Wen, 2011). Companies such as IBM, Huawei, and Alibaba have implemented mentorship programs for their employees. The mentor–protégé relationship is typically a developmentally oriented interpersonal relationship established between an experienced, senior individual and a less experienced, junior individual (Kram, 1985). Previous research has mainly focused on the effects of one-on-one mentoring on protégés’ career development, job performance, and affective aspects (Jie and Jian, 2015). However, as careers become increasingly borderless, mentoring relationships have evolved to become more networked and dynamic. According to social network theory, people’s behaviors are influenced by the social networks or structures they are embedded in; however, few studies have explored the mechanisms by which mentoring relationships influence employee creativity from a network perspective.

Studies have increasingly explored the effect of mentoring on employee creativity and the underlying mechanisms. For instance, research has shown that mentor relationships can enhance employees’ innovation potential by stimulating their work vitality and psychological security (Hu et al., 2020). However, there is limited exploration of the relationship between mentorship and innovation from a knowledge-management perspective. Knowledge is the foundation of innovation and is considered an essential strategic resource and core asset (Dong et al., 2019). Knowledge acquisition is the first step in the knowledge-management process (Nonaka and Takeuchi, 1995). In particular, tacit knowledge acquisition holds great value for employee creativity. Therefore, the role of tacit knowledge acquisition in the relationship between mentor network relationship strength and employee creativity warrants further exploration.

With rapid advancements in artificial intelligence (AI) technologies such as speech recognition, computer vision, and generative AI (e.g., ChatGPT), many organizations have adopted AI to drive their evolution toward greater intelligence. Given its potential to optimize decision-making and enhance efficiency, AI is empowering a wide range of industries and is being used in various application scenarios (Chowdhury et al., 2023). Amazon, for instance, has integrated AI with its workforce to enhance customer service. State Grid, meanwhile, utilizes AI in tandem with employees to identify equipment faults. According to the Asia-Pacific Artificial Intelligence Readiness Index 2023, 96% of small- and medium-sized enterprises are prepared to adopt AI. However, acquiring organizational innovation advantages does not depend solely on introducing smart technologies but on whether the roles played by human workers and AI in employee–AI interaction help leverage their respective strengths. When AI enhances human workers’ innovative behaviors rather than inhibiting them, it can become a powerful driver of organizational success (Makarius et al., 2020).

In this context, employee–AI collaboration is gradually becoming an important work model (Khoa et al., 2023; Kong et al., 2023). In such collaborations, AI helps employees deal with repetitive and mechanized work procedures, helping them focus more on creative tasks (Jia et al., 2024). Yin et al. (2024) found that employees, when collaborating with AI, experience an expansion of their work capacity, leading to improved innovative performance. Thus, similar to a workplace mentor, AI can also provide support to employees. However, there is limited research on the combined effect of employee–AI collaboration and mentor networks on employee creativity. This study, therefore, explores this issue, aiming to develop the value of employee–AI collaboration in interpersonal interactions to a greater extent. Specifically, we consider whether mentor network strength is better able to help employees acquire tacit knowledge and thus enhance employee creativity through employee–AI collaboration, as well as the role employee–AI collaboration plays in this process. In summary, we aim to address the following questions:

RQ1. In terms of knowledge management, how do mentor networks affect employee creativity? What is the underlying mechanism?

RQ2. What role does employee–AI cooperation play in the process of promoting employee creativity through mentor networks?

This study makes several contributions. First, we contribute to existing research by uncovering the relationship between mentor network strength and employee creativity. Second, this study reveals the mediating effect of cognitive and skill-based tacit knowledge acquisition on the mentor network strength–employee creativity relationship. This helps answer the question of how mentor networks affect employee creativity from a knowledge-management perspective. Third, based on conservation of resources theory, we explore the moderating effect of employee–AI collaboration on the relationship between mentor network strength and tacit knowledge acquisition. Our findings also contribute to a deeper understanding of how both interpersonal (mentor–protégé) and employee–AI collaborations influence employee knowledge acquisition and creativity. This provides valuable insights for stimulating employee creativity in the current context of digital intelligence.

## 2 Literature review and hypothesis development

### 2.1 Mentors and mentor networks

A mentor is defined as someone who is experienced and who provides professional and psychological support to protégés; this mentoring relationship is a single dyadic relationship (Kram, 1985). According to Scandura and Ragins (1993), mentors perform three functions—career support, psychosocial support, and role modeling—which are beneficial for employees’ positive behaviors. Previous studies have largely examined individual mentors, neglecting the mentor network level (Eby and Allen, 2002). Given the complexity of societies and interpersonal relationships, traditional mentoring relationships have evolved into network structures. It is more realistic, then, to study mentor theory from a network perspective (Ragins and Scandura, 1999). The mentor concept has evolved beyond the traditional “one-on-one” relationship to encompass a more collective notion, sometimes called a “mentor network” (Haggard et al., 2011).

Mentor network strength is an indicator that reflects the nature of these network relationships (Granovetter, 1973; Kram, 1985). It indicates the closeness of the relationships between mentors and protégés. Higgins and Kram (2001) also pointed out, strong ties provide relatively more psychosocial support than weak ties owing to emotional intimacy. Strong ties within a network lead to frequency of contact, reciprocity, and friendship (Granovetter, 1973). Therefore, the stronger the mentor network, the more frequent the interactions between protégés and mentors, which increases the likelihood of mentors helping protégés; this can predict the role of mentor network strength in promoting employee creativity. As a key indicator of mentor networks, mentor network strength is selected in this study to represent the mentor network structure to explore its role in promoting employee creativity. This responds to the suggestion by Dobrow et al. (2012) that it is essential to explore the influence of mentor networks in terms of their structural characteristics.

### 2.2 Mentor network strength and employee creativity

Creativity is defined as the ability to generate ideas or outcomes that combine novelty and utility (Amabile, 1996). Employee creativity, as a key driver of innovation, is influenced by a range of factors, including the knowledge, information, and resources available within an organization (Perry-Smith, 2006). Mentorship is an important tool for human-resource management and development in organizations, playing a crucial role in promoting employee development and gaining competitive advantage. In addition to guiding protégés’ vocational skills and career development, mentors provide psychosocial support and serve as role models (Ragins and Scandura, 1999). Thus, it has been suggested that mentoring relationships contribute to employees’ acquisition of essential skills and the provision of relevant resources, thereby enhancing their ability to engage in innovative activities (Liu et al., 2015).

Specifically, first, by providing career support, mentors play a crucial role in helping employees acquire job-specific knowledge, skills, and experience, thereby enhancing their cognitive capabilities and professional expertise (Kram, 1985). Access to necessary information and resources can stimulate employees to generate new ideas and explore new solutions to problems (Hennessey and Amabile, 2010). Second, mentors not only teach employees career skills so that they can quickly adapt to the work environment and organizational culture, but they also help reduce role ambiguity and conflict through psychological support (McManus and Russell, 1997). Third, by modeling effective behaviors, mentors serve as role models, enabling employees to imitate and internalize work behaviors, which enhances their professional skills and expands their work experience and practical skills (Wanberg et al., 2003). This, in turn, helps stimulate innovative thinking.

Social network theory suggests that the structural characteristics of the network an individual is embedded in, the location of the network, and the strength of the ties affect the information and social capital the individual acquires (Burt, 1992; Granovetter, 1973). Mentor network strength refers to the closeness of the protégé–mentor relationship, including the frequency of interaction, closeness, and mutual trust (Burt, 1992). The higher the relationship closeness between employees and mentors, the more it helps the mentor network exert a positive influence. We therefore propose the following:

*H*1. Mentor network strength has a positive effect on employee creativity.

### 2.3 Mentor network strength and tacit knowledge acquisition

Knowledge is a critical strategic resource for enhancing individual qualities and capabilities, playing a pivotal role in fostering creativity (Duan et al., 2020). In the knowledge economy, knowledge acquisition has received widespread attention as the foundation of knowledge management. It refers to the process by which individuals acquire new knowledge from external sources (Gang et al., 2017). According to Polanyi (1997), human knowledge can be broadly categorized into two types: explicit knowledge, which can be clearly articulated through language, numbers, symbols, or icons, and tacit knowledge, which cannot be systematically expressed but is embedded in actions and practices. Tacit knowledge is inherently personal, context-specific, and often difficult to formalize (Wang and Chang, 2017). Building on Nonaka and Takeuchi (1995) taxonomy, this study distinguishes between two forms of tacit knowledge acquisition: cognitive tacit knowledge acquisition and skill-based tacit knowledge acquisition. Cognitive tacit knowledge encompasses modes of thinking, cognitive styles, values, beliefs, and cultural norms; skill-based tacit knowledge acquisition involves experience, technology, skill, and management practices (Fan et al., 2014).

Nonaka and Takeuchi (1995) proposed the SECI model to describe the process of knowledge creation and transfer in an organization. This model presents two modes of tacit knowledge transfer: one is internalization, which is the process of transforming explicit knowledge into tacit knowledge, similar to “learning by doing”; and the other is socialization, which involves sharing experiences to create mental models and technical tools. The acquisition and transformation of tacit knowledge can be achieved through action learning and informal interactions among individuals (Koskinen et al., 2003). Informal learning, storytelling, and mentorship are the most effective ways to internalize and socialize tacit knowledge (Swap et al., 2001).

Through the sharing and transfer of knowledge between mentor and protégé, the mentor’s tacit knowledge is transmitted, which enhances the protégé’s professional competencies and ultimately contributes to improved organizational performance (Li et al., 2014). The higher the degree of mutual trust and the frequency of informal communication in employees’ willingness to share, the higher the likelihood of tacit knowledge transformation (Chin et al., 2024). It has been suggested that strong relationships promote trust and collaboration, thereby enabling individuals to acquire more nuanced, high-quality knowledge (Burt, 1992). This suggests that relationship quality is a crucial driver of tacit knowledge acquisition. Building on this, we hypothesize that stronger mentor network ties not only facilitate the acquisition of skill-based tacit knowledge (such as relevant experience and professional skills) but also contribute to the formation of culture-based tacit knowledge (such as values and cognitive frameworks). Therefore, we propose the following:

*H*2a. Mentor network strength has a positive effect on cognitive tacit knowledge acquisition.

*H*2b. Mentor network strength has a positive effect on skill-based tacit knowledge acquisition.

### 2.4 Mediating role of tacit knowledge acquisition

The essence of innovation is knowledge reorganization (Cohen and Levinthal, 1990). From a resource-based perspective, knowledge is a crucial determinant of organizational survival and growth, serving as a core resource that enables firms to establish competitive advantage (Grant, 1996). The main source of individual creativity is tacit knowledge, which is scarce, valuable, and difficult to imitate (Nonaka and Takeuchi, 1995).

Liu (2017) found that both cognitive tacit knowledge acquisition and skill-based tacit knowledge acquisition have a significant positive effect on entrepreneurial survival performance. Fan et al. (2014) showed that both organizational culture–based and rooted tacit knowledge acquisition (i.e., cognitive and skill-based tacit knowledge acquisition, as proposed in this study) can promote breakthrough innovation performance. Cognitive tacit knowledge acquisition reflects the similarity of values and innovation concepts of communicating subjects. This is more capable of reducing psychological distance, overcoming psychological barriers, and creating favorable conditions for innovation. Skill-based tacit knowledge acquisition focuses more on skill operation, and external knowledge accumulation can be acquired through different knowledge sources (Polanyi, 1997). As such, both cognitive tacit knowledge acquisition and skill-based tacit knowledge acquisition positively affect employee creativity. Thus, we propose the following:

*H*3a: Cognitive tacit knowledge acquisition has a significant positive effect on employee creativity.

*H*3b: Skill-based tacit knowledge acquisition has a significant positive effect on employee creativity.

Experienced mentors can provide targeted guidance to less experienced employees (Bang and Reio Jr, 2017). This process of experience exchange between mentors and protégés can continuously integrate, optimize, and upgrade the expertise of the protégé (Curtis and Taylor, 2018), which is an effective way to promote individual innovation (Hu et al., 2020). Social networks have significant effects on employees’ innovative capabilities (Burt, 1992), especially through knowledge exchange and emotional support within mentor networks, which can enhance employees’ creative thinking. In these networks, mentors help protégés think outside the box and open up new thinking spaces by transferring cognitive tacit knowledge, such as philosophy and values (Haggard et al., 2011), contributing to the emergence of innovation.

Mentors can impart technical and managerial skills to protégés, which is a highly valuable aspect of the mentor–protégé relationship (Uen et al., 2018). In a meta-analytical study, Eby and Allen (2002) found that protégés can develop professional skills through imitation, hands-on practice, and learning by doing within the mentor–protégé dynamic. Feedback and correction from the mentor are especially crucial for the employee’s skill development (Lankau and Scandura, 2002). We can infer from this that mentors help protégés acquire skill-based tacit knowledge through direct demonstration, feedback, and correction. The mastery of such knowledge forms a foundational base for innovation, as it enables protégés to refine their abilities and apply them creatively to problem-solving and idea generation. We therefore propose the following:

*H*4a: Cognitive tacit knowledge acquisition mediates the relationship between mentor network strength and employee creativity.

*H*4b: Skill-based tacit knowledge acquisition mediates the relationship between mentor network strength and employee creativity.

### 2.5 Moderating role of employee–AI collaboration

With the increasing integration of AI in the workplace, employee–AI collaboration has gradually emerged as a dominant work mode (Chowdhury et al., 2022). In research on the relationship between technology and work, traditional analytical perspectives have tended to view technology as either a “medium” (Bechky, 2003) or a “tool” (Nelson and Irwin, 2014). Anthony et al. (2023) proposed the systemic analytical perspective of employee–AI collaboration. Here, employee–AI collaboration refers to the process by which employees work together with AI (Sowa et al., 2021). In this context, AI is a collective term for software and hardware driven by AI technology that can assist or collaborate with humans in performing tasks (Moussawi et al., 2021). Such AI systems differ from AI-driven automated systems and algorithm management, which usually serve as assistants and colleagues in the workplace and collaborate with employees to complete tasks (Chin et al., 2024); thus, such AIs have also been referred to as AI assistants (Jia et al., 2024), AI colleagues (Savela et al., 2021), and AI team members (Seeber et al., 2020). Beyond these common intelligent “assistants,” this study also addresses embedded AI-driven software systems, which may lack a physical or virtual form of intelligence but still collaborate with employees. For example, AI assistants for online customer service (Jia et al., 2024) are equipped with simple question-and-answer and knowledge-gathering functions. Although AI takes diverse forms in the workplace, these systems share a common role in shaping how employees access, process, and apply knowledge (Anthony et al., 2023; Sowa et al., 2021; Moussawi et al., 2021). In this study, we conceptualize employee–AI collaboration as a work arrangement rather than a specific technological category. Accordingly, our focus is not on differentiating between specific AI types or technical architectures, but on capturing the collaborative interaction pattern between employees and AI.

Noting that employee–AI collaboration is an important resource situation, Bankins et al. (2024) called for analyzing the mechanism of action in employee–AI collaboration from a resource perspective. Drawing on Conservation of Resources Theory, individuals strive to protect, invest, and accumulate valuable resources, and situational conditions that facilitate individuals’ investment and utilization of available resources can amplify the effects of those resources on performance-related outcomes (Hobfoll et al., 2018). Therefore, we conceptualize employee–AI collaboration as a situational resource amplifier rather than a direct source of tacit knowledge. Tacit knowledge is inherently difficult to be systematically expressed and is primarily acquired through close interpersonal interaction, observation, and experiential learning (Polanyi, 1997). Although mentors remain the principal source of tacit knowledge, employees differ substantially in their capacity to absorb and transform tacit knowledge provide by their mentors.

Employee–AI collaboration enhances employees’ acquisition of tacit knowledge from their mentors through multiple mechanisms. First, previous studies have shown that AI has become one of the main channels for employees to access information, access technical resources (Del Giudice and Maggioni, 2014), and search for knowledge more quickly (Kemp, 2024), comprehensively (Wu et al., 2023), and accurately (Chowdhury et al., 2022). Employee–AI collaboration can help employees quickly search for and filter information, receive information support (Chowdhury et al., 2022), broaden knowledge-acquisition channels, and enrich *a priori* knowledge (Chin et al. 2023b; Chin et al. 2023a). From a conservation of resources perspective, employee–AI collaboration functions as a resource-supportive work condition that shapes how employees conserve and utilize their cognitive and learning resources. By reducing cognitive load associated with information searching, filtering, and routine decision-making, AI frees employees’ cognitive resources for sense-making and reflection on mentors’ guidance. At the same time, employee–AI collaboration facilitates the conversion of social resources into personal knowledge resources. Specifically, employee–AI collaboration reshapes learning environments by providing real-time informational support and facilitating capability development, which enables employees to more effectively process and internalize experience-based knowledge (Vrontis et al., 2022).

Second, in terms of the understanding and practice of complex knowledge structures, AI can also support employees’ acquisition of tacit knowledge through contextual simulations (Brynjolfsson and McAfee, 2014). This makes the mentor’s tacit knowledge explicit and provides situational validation. Moreover, when AI-enabled technologies are perceived as developmental opportunities, employees are more likely to invest cognitive and learning resources in adaptation and informal learning, thereby strengthening their ability to transform available knowledge resources into personal competencies (Ding, 2021; Xu et al., 2023). This suggests that when the level of employee–AI collaboration is high, individuals with adequate resources are more sensitive to the resources provided by the mentor network, and they are more likely to acquire cognitive and skill-based tacit knowledge from their mentors.

Consequently, when employee–AI collaboration is high, employees are better able to retain, integrate, and apply the tacit knowledge obtained from their mentor networks. Under such conditions, the same level of mentor network strength yields greater cognitive and skill-based tacit knowledge acquisition. In contrast, when employee–AI collaboration is low, higher cognitive constraints limit employees’ capacity to leverage mentor network resources effectively. Thus, we propose the following:

*H*5a: Employee–AI collaboration positively moderates the effect of mentor network strength on employees’ cognitive tacit knowledge acquisition.

*H*5b: Employee–AI collaboration positively moderates the effect of mentor network strength on employees’ skill-based tacit knowledge acquisition.

By combining H4a and H4b, we construct a moderated mediated effect model in which mentor network strength indirectly affects employee creativity by helping employees acquire cognitive and skill-based tacit knowledge. The magnitude of this indirect effect varies with the degree of employee–AI collaboration. Specifically, when the degree of employee–AI collaboration is high, AI reshapes the cognitive and learning context by reducing information-processing demands and supporting contextual interpretation. These conditions enhance employees’ ability to interpret, internalize, and apply tacit knowledge provided by mentors, thereby strengthening the conversion of mentor network resources into cognitive and skill-based tacit knowledge and amplifying the indirect effect of mentor network strength on employee creativity. In contrast, when the degree of employee–AI collaboration is low, employees face greater cognitive constraints and fewer learning supports which limits the efficiency of employees in absorbing and transforming the tacit knowledge provided by mentors. Consequently, the indirect effect of mentor network strength on employee creativity through tacit knowledge acquisition is weakened. Based on this, we propose the following:

*H*6a: Employee–AI collaboration positively moderates the indirect effect of mentor network strength on employee creativity through cognitive tacit knowledge acquisition.

*H*6b: Employee–AI collaboration positively moderates the indirect effect of mentor network strength on employee creativity through skill-based tacit knowledge acquisition.

**Figure 1:**
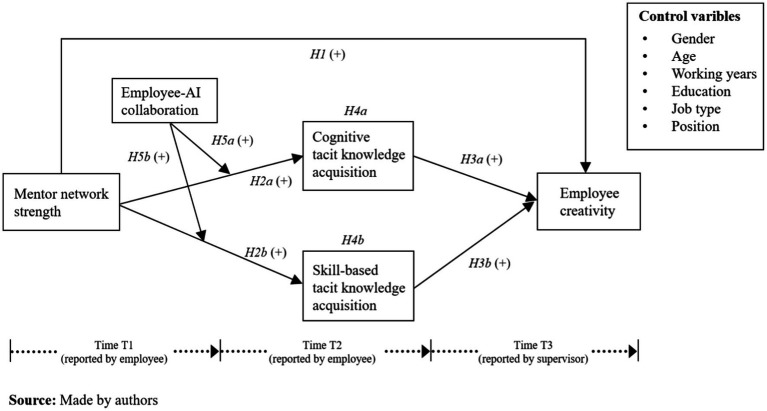
Theoretical framework.

Figure 1 illustrates this study’s theoretical model.

## 3 Method

### 3.1 Measures

All variables except mentor network strength used a five-point Likert-type scales (1 = completely disagree to 5 = completely agree). The scale used in this article is a mature scale published in authoritative academic journals in both Chinese and English. The scales in English were adapted and adopted after being translated into Chinese by two proficient bilingual translators through translation and back-translation procedures (Brislin, 1970).

#### 3.1.1 Mentor network strength

We use the egocentric network method to gather data. This approach involves using a name generator to identify mentors (Marsden, 1990). An egocentric network refers to the unique circle of social connections surrounding an individual. The study of egocentric networks aims to investigate correlations between an individual’s social interactions and variables analyzed at the individual level (Walker et al., 1993). We adopt two steps to measure the strength of the mentor network: identifying the mentors and calculating the tie strength of the mentor relationships.

First, we identify and select the mentors as the appropriate members of the mentor network. In line with previous research on egocentric networks (Ibarra, 1993; Mossholder et al., 2005), we provided a chart for the respondents. They were asked to fill in the first column with the initials of “people within their organization whom they view as sources of assistance when seeking information and advice related to their career.” Following Morrison (2002), we included eight rows for respondents to generate an initial list of up to eight contacts. The respondents then completed a section consisting of a matrix designed to evaluate relationships. We selected mentors based on the following two criteria: those (1) possessing a higher level of position and (2) providing career-related guidance. For the first criterion, respondents evaluated the position level of the nominated person and identified the person whose scores were greater than or equal to 3 (i.e., supervisor) as mentors. Following Dobrow and Higgins (2005), the second criterion was operationalized by asking the question, “How helpful was this nominated person in contributing to your professional advancement?” Ratings ranged from 1 (not at all) to 4 (very helpful). Mentors were identified as those who scored above 3 points (i.e., helpful).

Second, similar to Morrison (2002), after the selection of mentors, we assessed mentor network strength using the following question: “How emotionally close do you feel to this nominated person?” The response scale consisted of four points, ranging from 1 (distant) to 4 (especially close). Additionally, the mentor network strength score was determined by taking the average emotional intimacy score across all mentors and protégés (Morrison, 2002). The score ranges from 1 to 4.

#### 3.1.2 Employee–AI collaboration

We use the five-item scale developed by Kong et al. (2023) to evaluate employee–AI collaboration. An example item is “AI participates in my problem-solving process.” The Cronbach’s alpha is 0.804.

#### 3.1.3 Tacit knowledge acquisition

To measure cognitive tacit knowledge and skill-based tacit knowledge, we use a scale proposed by Fan and Wang (2011). We preceded this scale with detailed examples of cognitive and skill-based tacit knowledge. For example, cognitive tacit knowledge includes: mindset, organizational culture, beliefs and culture, philosophy, etc.; skill-based tacit knowledge includes: experience, technology, skills, etc.

The cognitive tacit knowledge acquisition scale comprises four items. Example: “I can acquire knowledge at the cognitive level promptly and accurately, including beliefs, values, organizational culture, etc.” The Cronbach’s alpha is 0.806.

The skill-based tacit knowledge acquisition scale comprises four items. Example: “I can acquire more knowledge at the technical level, including technology, skills, etc.” The Cronbach’s alpha is 0.827.

#### 3.1.4 Employee creativity

We use a four-item scale developed by Farmer et al. (2003). A sample item is “This employee often has innovative ideas.” The Cronbach’s alpha is 0.811.

#### 3.1.5 Controlled variables

Following previous studies (Dhar, 2016), we select common demographic variables—gender, age, working years, education level, job type, and position—as control measures to mitigate the potential effect of demographic disparities among employees on employee creativity.

### 3.2 Participants and procedures

Based on social relationships, we asked nine enterprises that use AI to distribute questionnaires. The survey respondents were staff members of these nine enterprises, located in Beijing, Hebei, Shenzhen, Anhui, and Shandong, among other areas. The industries of these enterprises included smart manufacturing, smart healthcare, smart business and retail, smart education, smart security, smart government affairs, and culture and media. In order to minimize the interference of common methodological bias on the research results, the data were collected in three-time periods, with an interval of 1 month between each distribution.

First, we communicated with a person in charge at each enterprise to explain the purpose of the research and ask for permission to conduct the study. With the permission of the superiors of each enterprise, we contacted the HR department of each enterprise and selected relevant contacts to help collect basic employee information.

Second, we ensured voluntary participation on the part of all respondents. We sent the questionnaire website link to our contacts at each enterprise, who subsequently forwarded the link to the participants via a WeChat or Ding Talk group. Respondents were initially queried about the existence of mentoring relationships in their organizations; only those who responded affirmatively were considered for the survey.

Third, we asked participants to fill in the last four digits of their personal or cell phone number and reminded them to be consistent in both phases (the third phase was filled in by the employee’s supervisor); otherwise, it was considered invalid. In addition, we followed Man Tang et al. (2022) to ensure that the participants distinguished AI technology from traditional technology (computers, Internet use, office tools such as Microsoft Word, Excel, etc.). The questionnaire gave a definition of AI technology in the guideline, i.e., it is an emerging technology with autonomous learning, reasoning, problem-solving, and decision-making abilities, including machine learning, intelligent recognition, and intelligent robotics. We included screening questions (e.g., “Does your job require the use of AI equipment or technology?”) to ensure that the participants were employees who actually work with AI. After completing the questionnaire, each subject received bonuses of varying amounts as a reward.

A total of 728 questionnaires were distributed at time point 1 to collect information on mentor network strength, employee–AI collaboration, and control variables; then, 615 valid questionnaires were recovered after excluding those with missing answers, time anomalies, and obvious patterns.

At time point 2, employees who participated in the previous stage were invited to fill out questionnaires to collect information on cognitive tacit knowledge acquisition and skill-based tacit knowledge acquisition; a total of 569 valid questionnaires were recovered.

At time point 3, employees’ direct supervisors were invited to evaluate employee creativity. A total of 523 valid questionnaires were recovered after data matching at three time points. After matching the three waves of data, a total of 523 valid questionnaires were obtained, for an effective recovery rate of 71.84%.

In this survey, employees aged 35 and below accounted for 91.6% of the total. Males made up 48.4%, while females accounted for 51.6%. Regarding tenure, 29.6% had 1–2 years of experience, 41.7% had 3–5 years, and 28.7% had over 5 years. Regarding education, 17.4% held junior college degrees or below, 70.7% had bachelor’s degrees, and 11.9% possessed postgraduate degrees and above.

## 4 Results

### 4.1 Common-method bias

We collected data at three time points and controlled for the confidentiality of the samples and the duration of responses to reduce common-method bias. To further examine common-method bias, Harman’s single-factor test is employed for factor analysis. The results reveal that the variance explained by the first factor is 26.74%, which is below the 40% threshold commonly suggested in previous research. This suggests that common-method bias is not severe in this study (Ashford and Tsui, 1991).

### 4.2 Validity analysis

#### 4.2.1 Discriminant validity analysis

Table 1 shows the results of discriminant validity analysis. The average variance extracted (AVE) root value of cognitive tacit knowledge acquisition is 0.797, which is greater than that for the relationship between cognitive tacit knowledge acquisition and skill-based tacit knowledge acquisition, employee–AI collaboration, and employee creativity (the maximum value is 0.365). The AVE root value of skill-based tacit knowledge acquisition is 0.811, which is greater than the value of the relationship between it and the other three factors (the maximum value is 0.414). Similarly, the AVE root value of employee–AI collaboration and the AVE root value of employee creativity are both greater than their correlation values with other factors, indicating good discriminant validity for the questionnaire measurement data.

**Table 1:** Discriminative validity analysis.

Latent variable	CTKA	STKA	EAIC	EC
CTKA	0.797			
STKA	0.365	0.811		
EAIC	0.013	0.300	0.753	
EC	0.357	0.414	0.193	0.800

The diagonal number is the AVE square root value; AVE, average variance extracted; CTKA, cognitive tacit knowledge acquisition; STKA, skill-based tacit knowledge acquisition; EAIC, employee-AI collaboration; EC, employee creativity.

#### 4.2.2 Confirmatory factor analysis

In this study, AMOS 26.0 was used for confirmatory factor analysis, with the results shown in Table 2. Compared with other models, the four-factor model shows good fit. (*χ*^2^ = 150.159, df = 113, *χ*^2^/df = 1.329, CFI = 0.988, NFI = 0.952, RESEA = 0.025) The results indicate that the scales demonstrate acceptable internal validity; thus, all scales are suitable for hypothesis testing with good discriminant validity.

**Table 2:** Confirmatory factor analysis of model fit.

Model	Factors	χ^2^	df	χ^2^/df	RESEA	CFI	NFI
Four-factor model	CTKA, STKA, EAIC, EC	150.159	113	1.329	0.025	0.988	0.952
Three-factor model	CTKA+STKA, EAIC, EC	680.331	116	5.865	0.097	0.812	0.783
Two-factor model	CTKA+STKA+EAIC, EC	1356.830	118	11.499	0.142	0.587	0.567
One-factor model	CTKA+STKA+EAIC+EC	1792.797	119	15.066	0.164	0.442	0.428

#### 4.2.3 Convergent and discriminant validity

We also examine the validity of the constructs in the final measurement model. As shown in Table 3, every construct consists of valid and reliable measurement items. Composite reliability (Crossan et al., 1995) surpasses the recommended threshold of 0.60, and AVE is 0.5 or greater (Fornell and Larcker, 1981). This demonstrates the convergent validity of the variables.

**Table 3:** Convergent and discriminant validity.

Construct	Item	Factor loading	CR	AVE
Cognitive tacit knowledge acquisition	CTKA1	0.784	0.874	0.635
	CTKA2	0.786		
	CTKA3	0.806		
	CTKA4	0.810		
Skill-based tacit knowledge acquisition	STKA1	0.830	0.885	0.658
	STKA2	0.826		
	STKA3	0.804		
	STKA4	0.785		
Employee-AI collaboration	EAIC1	0.772	0.867	0.567
	EAIC2	0.651		
	EAIC3	0.727		
	EAIC4	0.785		
	EAIC5	0.820		
Employee creativity	EC1	0.800	0.876	0.639
	EC2	0.808		
	EC3	0.784		
	EC4	0.806		

CR, composite reliability; AVE, average variance extracted.

### 4.3 Descriptive analysis

We used SPSS 22.0 to do the descriptive and correlation analysis. As shown in Table 4, the mentor network strength was positively correlated with cognitive tacit knowledge acquisition (*r* = 0.355, *p* < 0.01), skill-based tacit knowledge acquisition (*r* = 0.563, *p* < 0.01), and employee creativity (*r* = 0.396, *p* < 0.01). Cognitive tacit knowledge acquisition was positively correlated with employee creativity (*r* = 0.293, *p* < 0.01). And skill-based tacit knowledge acquisition was positively correlated with employee creativity (*r* = 0.338, *p* < 0.01). The correlation results provide preliminary evidence for hypothesis testing.

**Table 4:** Descriptive analysis of all variables.

Variable	1	2	3	4	5	6	7	8	9	10	11
1 Gender											
2 Age	0.034										
3 Working years	0.079	0.648^**^									
4 Education	0.064	0.053	0.029								
5 Job type	0.394^**^	0.057	0.034	0.071							
6 Position	−0.076	0.314^**^	0.332^**^	0.125^**^	0.011						
7 MNS	−0.081	0.074	0.080	−0.026	−0.090^*^	0.056					
8 CTKA	−0.012	0.057	0.087^*^	0.012	−0.037	0.023	0.355^**^	(0.81)			
9 STKA	−0.059	0.031	0.010	−0.025	−0.124^**^	−0.005	0.563^**^	0.300^**^	(0.83)		
10 EAIC	−0.038	0.024	0.007	0.000	−0.021	−0.036	−0.036	0.003*	0.258**	(0.80)	
11 EC	−0.057	0.227^**^	0.196^**^	0.077	−0.114^**^	0.185^**^	0.396^**^	0.293^**^	0.338^**^	0.164^**^	(0.81)
Mean	1.516	1.816	2.421	2.914	2.639	1.583	2.983	4.018	3.743	3.688	3.947
SD	0.500	0.635	1.189	0.615	1.434	0.779	0.582	0.600	0.687	0.554	0.568

*N* = 523. ^*^p < 0.05; ^**^p < 0.01, ^***^p < 0.001; Reliability coefficients for the scales are in parentheses along the diagonal.

### 4.4 Hypothesis tests

#### 4.4.1 Mediating effect test

The hierarchical regression model is constructed using SPSS 22.0. First, Table 5 shows that mentor network strength has a significant positive effect on employee creativity (*β* = 0.370, *p* < 0.001) and cognitive tacit knowledge acquisition (*β* = 0.351, *p* < 0.001). Cognitive tacit knowledge acquisition has a significant positive effect on employee creativity (*β* = 0.165, *p* < 0.001). Therefore, H1, H2a, and H3a are supported. In addition, the positive effect of mentor network strength on employee creativity is weakened by the addition of cognitive tacit knowledge acquisition, which initially indicates that cognitive tacit knowledge acquisition plays a mediating role between mentor network strength and employee creativity. Second, mentor network strength has a significant positive effect on skill-based tacit knowledge acquisition (*β* = 0.560, *p* < 0.001), and skill-based tacit knowledge acquisition has a significant positive effect on employee creativity (*β* = 0.171, *p* < 0.001). Therefore, H2b and H3b are supported. In addition, the positive effect of mentor network strength on employee creativity is weakened by the addition of skill-based tacit knowledge acquisition, which initially indicates that skill-based tacit knowledge acquisition plays a mediating role between mentor network strength and employee creativity. Thus, H4a and H4b are initially supported.

**Table 5:** The results of hierarchical regression model.

Variable	CTKA		STKA		EC			
	Model 1	Model 2	Model 3	Model 4	Model 5	Model 6	Model 7	Model 8
Constant	3.929^***^	2.819^***^	3.910^***^	1.886^***^	3.479^***^	2.373^***^	1.934^***^	2.107^***^
Gender	−0.006	0.014	−0.012	0.019	−0.014	0.007	0.005	0.004
Age	0.003	−0.011	0.053	0.031	0.157^**^	0.142^**^	0.144^**^	0.137^**^
Working years	0.090	0.071	−0.015	−0.045	0.063	0.043	0.031	0.051
Education	0.014	0.022	−0.017	−0.003	0.064	0.073	0.069	0.073
Job type	−0.039	−0.014	−0.121^**^	−0.081^**^	−0.125^**^	−0.099^*^	−0.097^*^	−0.085^*^
Position	−0.009	−0.018	−0.014	−0.028	0.107^*^	0.098*	0.101^*^	0.103^*^
MNS		0.351^***^		0.560^***^		0.370^***^	0.312^***^	0.274^***^
CTKA							0.165^***^	
STKA								0.171^***^
F	0.820	11.021^***^	1.551	35.419^***^	8.257^***^	20.951^***^	20.884^***^	20.449^***^
R^2^	0.009	0.130	0.018	0.325	0.088	0.222	0.245	0.241
∆R^2^	0.002	0.118^***^	0.006	0.316^***^	0.077^***^	0.211^***^	0.234^***^	0.230^***^

*N =* 523. ^*^p < 0.05; ^**^p < 0.01, ^***^p < 0.001.

Following Hayes (2013), we use the Process 4.1 plug-in in SPSS for bootstrapping to further verify the mediating effect. As shown in Table 6, the total effect of mentor network strength on employee creativity is significant because the 95% confidence interval excludes 0. The direct effect of the mentor network on employee creativity is significant because the 95% confidence interval excludes 0. Mentor network strength has a significant positive effect on employee creativity through cognitive tacit knowledge acquisition. Its 95% confidence interval does not contain 0, suggesting that cognitive tacit knowledge acquisition plays a partial mediating role. Thus, H4a is further verified.

**Table 6:** Bootstrapping mediation testing results of cognitive tacit knowledge acquisition.

Pathway	Effect	SE	95% CI	
			Low	High
Total effect	0.361	0.038	0.285	0.436
Direct effect	0.304	0.040	0.225	0.383
MNS → CTKA→KS	0.056	0.017	0.026	0.092

CI, confidence interval.

As shown in Table 7, the total effect of mentor network strength on employee creativity is significant because the 95% confidence interval excludes 0. The direct effect of mentor network strength on employee creativity is significant because the 95% confidence interval excludes 0. Mentor network strength has a significant positive effect on employee creativity through skill-based tacit knowledge acquisition. Its 95% confidence interval does not contain 0, suggesting that skill-based tacit knowledge acquisition plays a partial mediating role. Thus, H4b is further verified. Moreover, the mediating effect of cognitive tacit knowledge acquisition is smaller than that of skill-based tacit knowledge acquisition.

**Table 7:** Bootstrapping mediation testing results of skill-based tacit knowledge acquisition.

Pathway	Effect	SE	95% CI	
			Low	High
Total effect	0.361	0.038	0.285	0.436
Direct effect	0.267	0.046	0.178	0.357
MNS → STKA→KS	0.093	0.031	0.029	0.154

CI, confidence interval.

#### 4.4.2 Moderating effect test

Using SPSS 22.0, we constructed a hierarchical regression analysis to empirically test the moderating effect. Table 8 presents the results. The results of Models 1 and 2 show that the interaction of mentor network strength and employee–AI collaboration has a significant positive effect on cognitive tacit knowledge acquisition (*β* = 0.195, *p* < 0.001). This indicates that employee–AI collaboration can positively moderate the effect of mentor network strength on cognitive tacit knowledge acquisition. Therefore, H5a is supported. Models 3 and 4 show that the interaction of mentor network strength and employee–AI collaboration has a significant positive effect on skill-based tacit knowledge acquisition (*β* = 0.131, *p* < 0.001). This indicates that employee–AI collaboration positively moderates the effect of mentor network strength on skill-based tacit knowledge acquisition. Therefore, H5b is supported. In addition, employee–AI collaboration plays a stronger moderating role between mentor network strength and skill-based tacit knowledge acquisition than between mentor network strength and cognitive tacit knowledge acquisition.

**Table 8:** Results of the moderation effects.

Variable	CTKA		STKA	
	Model 1	Model 2	Model 3	Model 4
Constant	2.759^***^	2.578^***^	0.563^*^	0.423
Gender	0.014	0.01	0.031	0.028
Age	−0.011	−0.008	0.019	0.022
Working years	0.071	0.068	−0.046	−0.047
Education	0.022	0.029	−0.005	0.001
Job type	−0.014	−0.001	−0.078*	−0.07
Position	−0.017	−0.029	−0.014	−0.022
MNS	0.352^***^	0.387^***^	0.571^***^	0.595^***^
EAIC	0.015	0.029	0.277^***^	0.287^***^
MNS × EAIC		0.195^***^		0.131^***^
F	9.642^***^	11.414^***^	43.066^***^	40.894^***^
R^2^	0.130	0.167	0.401	0.418
∆R^2^	0.117^***^	0.152^***^	0.392^***^	0.408^***^

*N =* 523. ^*^p < 0.05; ^**^p < 0.01, ^***^p < 0.001.

#### 4.4.3 Moderated mediating effect test

To further test the moderated mediating effects, we use the Process 4.1 plug-in to conduct bootstrap analysis in Model 7. As shown in Table 9, if employee–AI collaboration is low, then the effect is 0.034 (95% bias-corrected CI = [0.013, 0.061]; SE = 0.012), which does not include 0. If employee–AI collaboration is high, then the effect is 0.091 (95% bias-corrected CI = [0.043, 0.148]; SE = 0.027), which does not include 0. In addition, the effect of the differences between the high and low levels is 0.057 (95% bias-corrected CI = [0.022, 0.102]; and SE = 0.021), which does not include 0. This indicates that the moderated serial mediation effect of employee–AI collaboration is significant. Therefore, H6a is supported. Similarly, if employee–AI collaboration is low, then the effect is 0.079 (95% bias-corrected CI = [0.024, 0.133]; SE = 0.028), which does not include zero. If employee–AI collaboration is high, then the effect is 0.119 (95% bias corrected CI = [0.036, 0.197]; SE = 0.041), which does not include 0. Finally, the effect of the differences between the high and low levels is 0.040 (95% bias-corrected CI = [0.009, 0.080]; and SE = 0.018). This shows that the moderated serial mediation effect of employee–AI collaboration is significant. Therefore, H6b is supported.

**Table 9:** Moderated mediation effect of employee-AI collaboration.

Path	Moderating variable status	Effect	SE	95% CI	
				Low	High
MNS-CTKA-KS	Low EAIC	0.034	0.012	0.013	0.061
	High EAIC	0.091	0.027	0.043	0.148
	Difference group	0.057	0.021	0.022	0.102
MNS-STKA-EC	Low EAIC	0.079	0.028	0.024	0.133
	High EAIC	0.119	0.041	0.036	0.197
	Difference group	0.040	0.018	0.009	0.080

CI, confidence interval.

**Figure 2:**
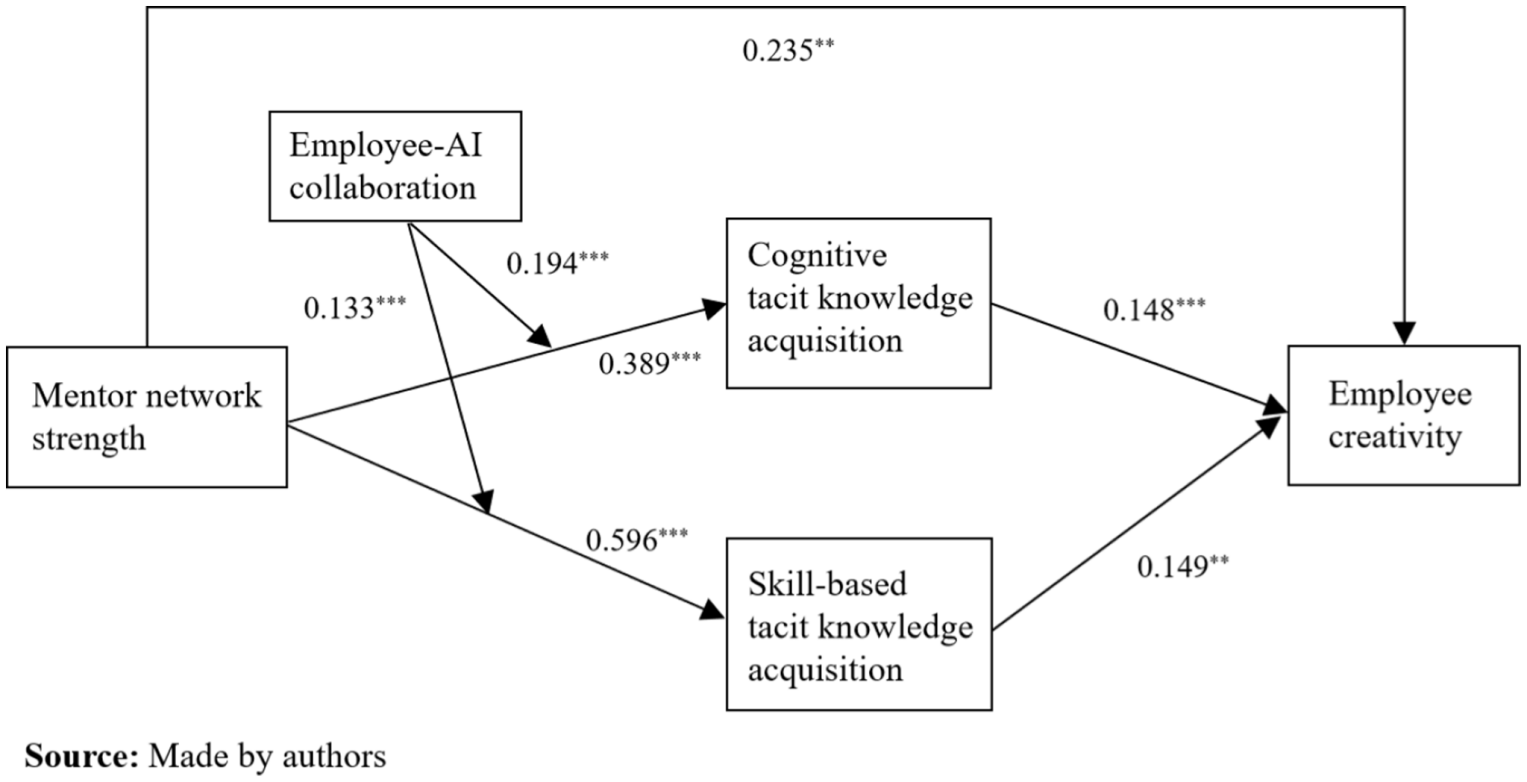
Path analysis result.

To verify the above test results, we use structural equation modeling to draw a model path analysis diagram, as shown in Figure 2. The path analysis results once again confirm the above test results.

**Figure 3:**
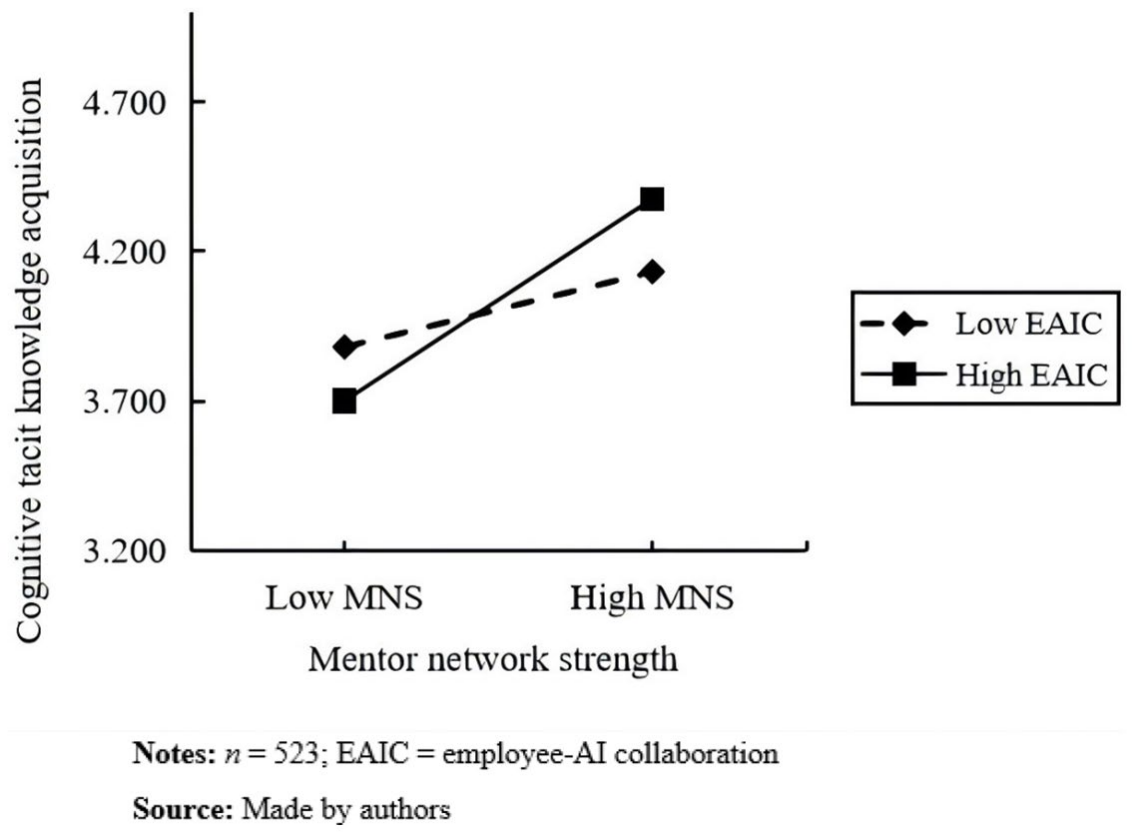
The moderating effect of EAIC on MNS and CTKA.

**Figure 4:**
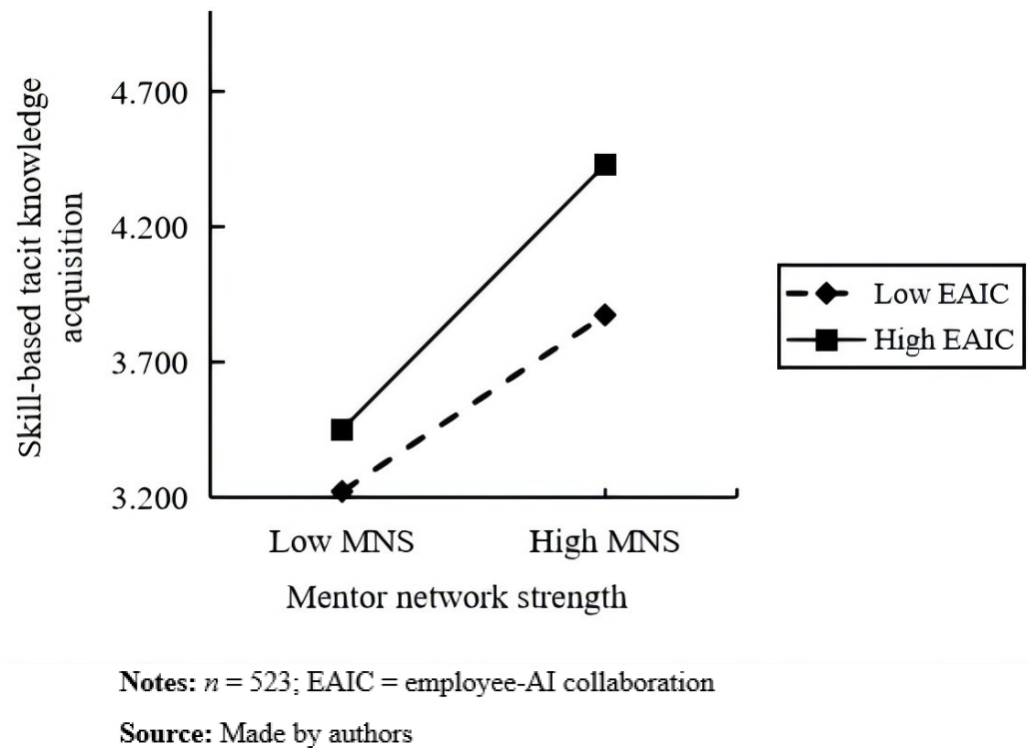
The moderating effect of EAIC on MNS and STKA.

Next, we further illustrate the moderating effects of employee–AI collaboration on the mentor network strength–cognitive tacit knowledge acquisition relationship, as well as the mentor network strength–skill-based tacit knowledge acquisition relationship. To do this, we used the simple slope analysis method of Aiken and West (1991) to draw moderating effect diagrams for employees with employee–AI collaboration above and below the mean by one standard deviation. As shown in Figure 3, under the high condition of employee–AI collaboration, employees’ mentor network strength has a stronger influence on cognitive tacit knowledge acquisition (simple slope = 0.58, *p* < 0.001). Under the low condition of employee–AI collaboration, the mentor network has a weaker influence (simple slope = 0.22, *p* < 0.001), i.e., employee–AI collaboration positively moderates the relationship between employees’ mentor network strength and cognitive tacit knowledge acquisition. Figure 4 shows that the influence of mentor network strength on skill-based tacit knowledge acquisition is stronger under a high degree of employee–AI collaboration (simple slope = 0.84, *p* < 0.001) and weaker under a low degree of employee–AI collaboration (simple slope = 0.56, *p* < 0.001); that is, with an increase in employee–AI collaboration, the positive effect of mentor network strength on skill-based tacit knowledge acquisition is gradually enhanced. Employee–AI collaboration positively moderates the relationship between mentor network strength and skill-based tacit knowledge acquisition.

## 5 Discussion

Human–AI collaboration in the workplace is now a major trend. While researchers continue to debate whether AI replaces or enhances employees (Raisch and Krakowski, 2021; Khoa et al., 2023), in reality, the large-scale replacement of employees by AI is not actually taking place. Rather, AIs participate in employees’ work as “partners” or “teammates,” and employee–AI collaboration has become an important work mode (Chowdhury et al., 2023). In this context, focusing on knowledge management, we explore the mechanism of employee–AI cooperation in the influence of mentor network strength on employee creativity. The conclusions are summarized below.

First, from a knowledge-management perspective, we explore the effect of mentor network strength on employee creativity, in which cognitive tacit knowledge acquisition and skill-based tacit knowledge acquisition mediate that effect. In particular, skill-based tacit knowledge acquisition plays a stronger mediating role than cognitive tacit knowledge acquisition. This could be because skill-based tacit knowledge is more operational, and the transformation process is more intuitive, which can directly contribute to the development of creativity through practice, imitation, and feedback (Polanyi, 1997). In addition to imparting experience and knowledge, mentors directly transfer work skills to their protégés through demonstration, practice, and feedback. Moreover, by providing sponsorship, protection, exposure, and visibility, mentors can also give their protégés opportunities to acquire formal and informal work-related knowledge. These opportunities not only enhance protégés’ practical skills but also foster their creative thinking (Baranik et al., 2017; Uen et al., 2018). However, cognitive tacit knowledge (e.g., values, beliefs) involves abstract thinking, judgment criteria, and decision-making logic (Nonaka and Takeuchi, 1995) and requires a longer period of learning, reflection, and introspection. Its effect on creativity is achieved more through indirect changes in thinking patterns and judgment. Thus, skill-based tacit knowledge acquisition plays a stronger mediating role between mentor network strength and employee creativity. This finding contributes to our understanding of the pathways through which mentoring influences employee creativity.

Second, based on conservation of resources theory, we demonstrate that employee–AI collaboration, as a significant situational resource in the workplace, positively moderates the relationship between mentor network strength and cognitive and skill-based tacit knowledge acquisition. Additionally, the indirect effects of mentor network strength on employee creativity via cognitive and skill-based tacit knowledge acquisition are strengthened by employee–AI collaboration. These findings suggest that employee–AI collaboration enhances the process of acquiring tacit knowledge from the mentor network. By providing supplementary resource support, reducing cognitive load, lowering the burden of information retrieval and processing (Petzsche et al., 2023), and alleviating psychological stress (Chowdhury et al., 2023), employee–AI collaboration enables employees to more efficiently acquire cognitive and skill-based tacit knowledge from their mentor network. These findings offer new insights and perspectives that can help organizations understand how employee–AI cooperation promotes employee creativity through mentor networks.

### 5.1 Theoretical implications

First, our study clarifies the mechanism by which mentor network strength influences employee creativity. This responds to Dobrow et al. (2012), who called for examining mentor relationships from a network perspective, thus further enriching mentor theory. While weak-tie and structural-hole theories emphasize the value of loosely connected relationships for accessing novel information and heterogeneous resources (Granovetter, 1973; Burt, 1992; Seibert et al., 2001), our findings suggest that strong ties with mentors are more critical when the focal outcome involves tacit knowledge acquisition, which requires trust, closeness, and sustained interaction. In the Chinese organizational context, where “guanxi” (relationship) is regarded as an important job resource (Wu et al., 2019), strong ties facilitate the transfer of experience-based knowledge and thereby enhance creativity. It can help us to better understand these theories’ applicability in different cultural contexts. In addition, some technology-oriented perspectives suggest that artificial intelligence may increasingly automate or substitute for certain cognitive and professional tasks, thereby reshaping the role of human expertise in decision-making processes (Brynjolfsson and McAfee, 2014). In contrast, our findings indicate that employee–AI collaboration primarily serves a complementary and amplifying function rather than a substitutive one.

Second, we expand research on tacit knowledge acquisition in the knowledge-management field and the practical application of knowledge-management theory. Previous studies have mainly explored the path of mentorship’s influence on employee creativity in terms of cognition or emotion (Hu et al., 2020) while neglecting employees’ knowledge. Knowledge acquisition is the first step in the knowledge-management process, which provides the basis for subsequent knowledge storage, sharing, application, and innovation (Nonaka and Takeuchi, 1995). In particular, the acquisition of tacit knowledge is highly valuable for enhancing employees’ innovative capabilities. From a knowledge-management perspective, this study illuminates the “black box” of the relationship between mentor network strength and employee creativity. We also explore the effects of different types of tacit knowledge acquisition, providing a framework for distinguishing the roles of different types of tacit knowledge in mentor networks’ promotion of innovation.

Third, this research provides a new theoretical perspective on the role of employee–AI collaboration in the workplace. Based on conservation of resources theory, we demonstrate that employee–AI collaboration acts as a situational resource that enhances the acquisition of tacit knowledge resources from the mentor network, ultimately fostering increased employee creativity. This responds to Bankins et al. (2024), who called for analyzing employee–AI collaboration from a resource perspective, providing new insights into how employee–AI collaboration functions in the tacit knowledge acquisition process. While previous studies have largely focused on the direct effect of employee–AI collaboration on employees or organizations (Chowdhury et al., 2022), we go further by addressing the need to explore the moderating role of employee–AI collaboration (Kong et al., 2023). This not only highlights the value of AI as a moderating factor but also deepens our understanding of how AI supports the broader dynamics of knowledge acquisition and creativity in the workplace. In this way, we take a step forward in exploring the moderating effect of employee–AI collaboration.

### 5.2 Practical implications

First, we confirm that strong mentor network strength can promote employee creativity. Managers should therefore attach importance to the role of mentoring in promoting employee creativity. They could establish talent development and training models based on mentoring relationships and ensure sustainable mentoring through institutionalization, such as formulating guidance plans and implementing a mentor training system to enhance mentors’ guidance capabilities. The exchange of experience between mentors and protégés facilitates the development of protégés’ innovative thinking. Therefore, managers should establish communication platforms to facilitate knowledge exchange and emotional cultivation between mentors and protégés, thereby strengthening their relationships, promoting the acquisition of tacit knowledge, and enhancing employee creativity.

Second, this study verifies that mentor network strength enhances employee creativity through tacit knowledge acquisition and identifies differences in the mediating roles played by different types of tacit knowledge. These findings can help organizations understand the mechanisms through which mentors promote employee creativity from a knowledge-management perspective. More effective systems and strategies for promoting the acquisition and transfer of tacit knowledge could be designed. Organizations should, for instance, pay attention to managing employees’ tacit knowledge and broadening the channels of tacit knowledge acquisition (e.g., using a system to collect potential tacit knowledge). They should also focus on employees’ intrinsic motivation, incorporate tacit knowledge into performance appraisals, prompt employees to obtain external resources to compensate for knowledge deficiencies, encourage employees to improve their knowledge structures, and stimulate innovative employee behavior. Further, building an open, trusting cultural atmosphere can help employees acquire and share tacit knowledge.

Third, we verify that employee–AI collaboration positively moderates the mentor network strength–tacit knowledge acquisition relationship. This provides new evidence for the positive effects of employee–AI collaboration, helping firms understand how human–AI collaboration can enhance the utility of interpersonal relationships for employees. When the rapid enhancement of employee knowledge becomes an organizational priority, firms should not only create traditional learning environments to facilitate knowledge transfer—such as mentorship programs and training sessions—but also enhance the efficiency of tacit knowledge acquisition by encouraging employees to collaborate with AI and designing employee–AI collaboration work models. Meanwhile, managers should implement strategies to optimize employee–AI collaboration. They should encourage employees to embrace digital tools and technologies, guide them in developing an accurate understanding of AI, and help them assess and respond to the effects of employee–AI collaboration in the workplace. Organizations should also take steps to foster harmonious human–AI relationships. This can be achieved by offering diverse, comprehensive AI-driven knowledge promotion and training initiatives, encouraging employees to participate in the iterative development of intelligent systems, positioning AI as an ideal partner for employees, and cultivating a collaborative human–machine culture within the organization.

### 5.3 Limitations and future research

First, this study did not take into account the influence of cultural context factors on the mentor network, and overlooked the impact of cultural factors such as the power distance and differential pattern between mentors and protégés on employee creativity. We conducted our research in a culture of high power distance and collectivism, Compared with a low power-distance culture, strong ties with higher -status people in a high power-distance culture might be more beneficial to knowledge and resource acquisition of employees. Future studies can investigate whether the effects we found differ in cultures of low power distance or individualism. Furthermore, research could be extended to a broader range of industries to enhance the generalizability of the findings.

Second, our sample is skewed toward younger employees, which reflects the demographics of digitally transforming Chinese firms but constrains the applicability of our conclusion. Controlling for age and tenure mitigates confounding but does not eliminate concerns about external validity or potential age-related heterogeneity. Consequently, the generalizability of our results to older employees—who may differ in cognitive styles, technology acceptance, and mentorship engagement—should be interpreted with caution.

Finally, we draw upon Morrison (2002) research and quantify the tie strength of mentors by considering the degree of emotional closeness as a single-dimensional indicator of relationship strength. Although the degree of emotional intimacy is a crucial factor in theoretical aspects for the transfer of tacit knowledge, future research can adopt multi-dimensional network measurement standards (such as interaction frequency or complexity) to further verify the reliability of the current research results. Moreover, this study exclusively focuses on the tie strength as a representation of the structural variables in the mentor network to carry out research. However, other structural variables such as centrality and density of mentor network are also worthy of further exploration in the future.

## 6 Conclusion

This study examines how mentor network strength enhances employee creativity through tacit knowledge acquisition and highlights the moderating role of employee–AI collaboration. The results indicate that mentor network strength promotes employee creativity through both cognitive and skill-based tacit knowledge acquisition, and skill-based tacit knowledge plays a more influential mediating role. Employee–AI collaboration positively moderates the relationship between mentor network strength and cognitive and skill-based tacit knowledge acquisition. Additionally, the indirect effects of mentor network strength on employee creativity via cognitive and skill-based tacit knowledge acquisition are strengthened by employee–AI collaboration. These findings provide new insights into how human and AI resources can jointly foster employee creativity.

## Data Availability

The datasets presented in this study can be found in online repositories. The names of the repository/repositories and accession number(s) can be found at: DOI: https://doi.org/10.6084/m9.figshare.30606050.
